# Non-Contact Respiration Measurement Method Based on RGB Camera Using 1D Convolutional Neural Networks

**DOI:** 10.3390/s21103456

**Published:** 2021-05-15

**Authors:** Hyeon-Sang Hwang, Eui-Chul Lee

**Affiliations:** 1 Department of Computer Science, Graduate School, Sangmyung University, Seoul 03016, Korea; hyeonsang92@naver.com; 2 Department of Human-Centered Artificial Intelligence, Sangmyung University, Seoul 03016, Korea

**Keywords:** biomedical monitoring, remote sensing, respiration, respiration rate, image processing, 1D convolution

## Abstract

Conventional respiration measurement requires a separate device and/or can cause discomfort, so it is difficult to perform routinely, even for patients with respiratory diseases. The development of contactless respiration measurement technology would reduce discomfort and help detect and prevent fatal diseases. Therefore, we propose a respiration measurement method using a learning-based region-of-interest detector and a clustering-based respiration pixel estimation technique. The proposed method consists of a model for classifying whether a pixel conveys respiration information based on its variance and a method for classifying pixels with clear breathing components using the symmetry of the respiration signals. The proposed method was evaluated with the data of 14 men and women acquired in an actual environment, and it was confirmed that the average error was within approximately 0.1 bpm. In addition, a Bland–Altman analysis confirmed that the measurement result had no error bias, and regression analysis confirmed that the correlation of the results with the reference is high. The proposed method, designed to be inexpensive, fast, and robust to noise, is potentially suitable for practical use in clinical scenarios.

## 1. Introduction

Medically, the measurement of respiration is an important indicator that enables the detection of serious human diseases before they become advanced. In fact, it has been reported that respiration information can be used as a predictor of chronic heart failure, cardiopulmonary arrest, and pneumonia [1,2,3]. However, although the measurement of respiration is highly important, routine breathing monitoring is not used, even when the patient’s main disease is respiratory abnormality [4]. This is because existing methods are inconvenient. Currently, the most commonly used respiration measurement methods include manual counting, measurement of the carbon dioxide concentration in a patient’s oxygen supply, or the attachment of a belt or electrode to detect movement [5]. The need for external assistance and/or the discomfort of attaching extra equipment to the body makes routine breathing monitoring difficult. Convenient and routine breathing measurement without the need for a separate device could help reduce various risks by detecting signs of serious disease in advance.

Methods that measure respiration remotely using a camera instead of an attached sensor have been investigated. These approaches can be divided into main three categories: thermal-camera-based methods, remote photoplethysmography (PPG)-based methods, and motion-based methods.

Hu et al. (2017) calibrated an RGB camera and a thermal camera, which makes it relatively easy to detect facial feature points. They detect the nostril region in the thermal image and observe the change in temperature of the nostril due to respiration [6]. Other studies such as Cho et al. (2017) use a method of tracking the nostril region using the gradients in thermal images [7]. Thermal-imaging-based methods are very robust methods that are not affected by changes in illumination as long as the nostril region of interest (ROI) is stably detected. However, high-resolution thermal imaging cameras are very expensive, and Hu (2018) showed that the performance of this approach decreases substantially when the resolution of a thermal image is 320 × 240 or less. Hence, it is a difficult method to use in general [8].

As an alternative, other methods use the changes in blood flow caused by breathing, and for this purpose, changes in skin color such as facial areas are tracked [9,10]. These methods should be robust to motion through a combination of techniques that detect facial areas, which function as representative skin areas. However, the observation of changes in blood flow due to respiration is not suitable for general use because, as Nam et al. (2014) found, the accuracy is greatly reduced for rapid breathing of 26 bpm or more [11].

Reyes et al. (2016) and Massaroni et al. (2019) observe the variance in pixels by manually selecting chest and neck regions as ROIs for measuring respiration through movement of the human body [12,13]. Bartula et al. (2013) also manually select an ROI and detect motion using a one-dimensional profile [14]. However, the manual selection of an ROI is a cumbersome task, and ROI reassignment is required or measurement is impossible if the subject moves and leaves the ROI.

To avoid the need to manually specify an ROI, attempts have been made to measure respiration with automatically detected ROIs. Wiede et al. (2017) and Ganfure et al. (2019) both detect face regions and then predict the body region accordingly [15,16]. In these methods, even if the body region is in the image, if the face cannot be detected, respiration cannot be measured, and even if the face is detected, the region necessary for respiration measurement may not be included in the ROI depending on the position of the body. As an alternative, Tan et al. (2010) analyze respiration by detecting a moving region in an image using the difference between adjacent frames [17]. In addition, Li et al. (2014) estimate the ROI based on the deviation of the motion trajectory of subregions [18]. These methods can work well when the subject is alone against a static background, but because they analyze the motion of the entire image, they are susceptible to noise caused by objects moving in the background.

Another approach was proposed by Janssen et al. (2016) [19]. This method detects the ROI by obtaining a motion matrix and assigning a score regarding the breathing characteristics for each pixel. This method is robust against the aforementioned problems, but it is difficult to measure respiration in real time because of the computational load of the Brox et al. dense optical flow method used for motion calculation [20].

Therefore, in this paper, to address these problems and enable practical general respiration measurement, we propose a method of estimating respiration based on an RGB webcam without the need for a separate device. Our research has three contributions: (1) Real-time performance is achieved by a camera-based method that does not use a separate, expensive device. (2) The entire process of the proposed respiration measurement is automated. (3) Using ROI detection and pixel selection using the characteristics of respiration, it is possible to measure respiration in a way that is robust to noise. We demonstrate the performance of the proposed method with respect to the conventionally measured reference signal.

## 2. Materials and Methods

**Figure 1:**
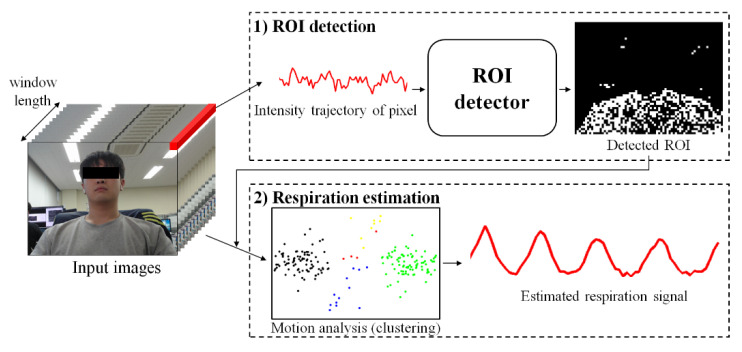
Overall process of the proposed method.

As shown in Figure 1, the proposed method consists of two steps: (1) ROI detection and (2) respiration estimation. The first step is to detect ROI related to respiration by classifying whether respiration information is included in the variance of pixels using a learning-based model. The second step is to remove noise by analyzing the variance of each pixel included in the ROI and estimate clear breathing information. Detailed descriptions of each step are provided in Section 3.1 and Section 3.2, respectively.

### 2.1. Detecting the ROI Using Machine Learning

**Figure 2:**
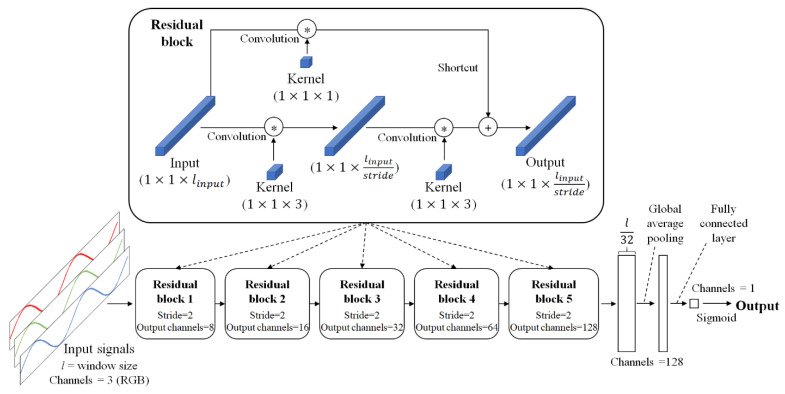
Structure of the ROI detection model. The variance in pixels is the input and the probability that the variance is related to respiration is the output.

The method proposed in this paper uses a simple classifier that acquires an image signal over a time window and classifies whether the variance in each pixel contains respiratory information, as shown in Figure 2.

Images used in the calculation are downsampled to reduce noise and the amount of computation. Moreover, the input signal (*l*) is at least twice as long as the maximum breathing period. We assumed a normal breathing range of 10 to 40 bpm to cover this, a range suggested in Hu [6], so the time window is more than 12 s long. The input signals contain red, green, and blue components. The model is composed of residual blocks and includes the shortcut mechanism of the ResNet architecture for fast optimization and performance improvement [21]. Here, each residual block consists of 1D convolution performed on the time series of the input signal instead of the 2D convolution used to extract spatial features. The kernel size of all convolutions is 3, and batch normalization and rectified linear unit activation functions are applied after each convolution. Each block first performs a convolution with a stride of 2 to replace the pooling layer. The extracted features become a 128-dimensional vector using global average pooling, and the output of the model is generated through a fully connected layer applying batch normalization and sigmoid. For training, binary cross-entropy is used as the loss function.

The ROI detector should be able to detect pixels containing the movements caused by breathing, regardless of spatial characteristics such as the subject’s location, appearance, or gender. Because the proposed model is limited so that spatial information cannot be used and only time-series information is used for inference, classification focusing on the variance in pixels is possible. This greatly reduces the amount of information that the model has to handle, allowing a lightweight model to be retained, and thereby enabling real-time computation. This also makes learning easier because a sufficiently large amount of data to train a complex model is not required.

### 2.2. Labeling Method for Model Training

To train the proposed model, input images and the corresponding pixel-level labels are required. However, it is very difficult and time consuming to manually classify each pixel containing the characteristics of respiration in a video. Therefore, in the approach proposed this paper, the following method is used to automatically perform labeling.

Because the purpose of the proposed model is to classify pixels containing respiration information, calculating the similarity between the reference respiration signal and the variance of each pixel can determine how close the pixel is to one with respiration information. To calculate the similarity, we use the Pearson correlation coefficient (*r*) as follows:

(1) $$r=\frac{\sum_{i=1}^{n}\left(v_{i}-\bar{v})(u_{i}-\bar{u}\right)}{\sqrt{\sum_{i=1}^{n}{\left(v_{i}-\bar{v}\right)}^{2}}\sqrt{\sum_{i=1}^{n}{\left(u_{i}-\bar{u}\right)}^{2}}}.$$

In the equation, *u* and *v* are the vectors used to calculate *r*, and in this case, they denote the reference signal and the trajectory of change in one arbitrary pixel (time window length), respectively. The value of *r* ranges from −1 to 1, and the closer it is to 0, the lower the correlation. Because a change in pixel value just indicates a change in color, it does not specify the direction of movement. Therefore, among the pixel change signals, there are also signals that are antiphase with respect to the reference signal. For the in-phase signals, *r* is closer to 1, and for the antiphase signals, *r* is closer to −1 as the respiration information becomes clearer. Therefore, the label for a pixel at position (*x*, *y*) of the image can be defined as follows for a specific threshold (*T*):

(2) $${label}_{x,y}=\{\begin{array}{cc} 1 & if\;\left|r_{x,y}\right|\geq T\\ 0 & otherwise \end{array}.$$

**Figure 3:**
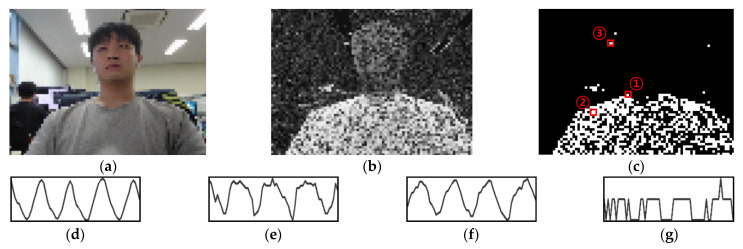
Labeling process for video using the reference and the variance in some pixels: (**a**) sample of a downsampled video; (**b**) correlation coefficient calculated from the video and reference signal; (**c**) label for the video (*T* = 0.7); (**d**) reference signal; (**e**–**g**) variance of Pixels 1–3, respectively.

Figure 3 shows the correlation coefficient, label, and several samples of the pixel variance determined in this way. For the actual respiration signal in Figure 3d, Figure 3e,f show the pixel-change signals with in-phase and antiphase variance, respectively. Using the proposed labeling method, most of the breathing pixels in the video can be automatically classified. However, as shown in Figure 3g, there may be cases in which noise pixels that have signals similar to respiration signals are accidentally misclassified. This misclassification is not a big problem for learning itself because there are few such pixels in the whole video. However, this suggests that in the single pixel-based classification method, there may be noise with a pattern that is difficult to distinguish from the respiratory signal. Therefore, so that the proposed model can be practically used, it is necessary to be able to distinguish between breathing pixels and noise pixels in a noisy ROI.

### 2.3. Estimating Respiration through Motion Analysis

**Figure 4:**
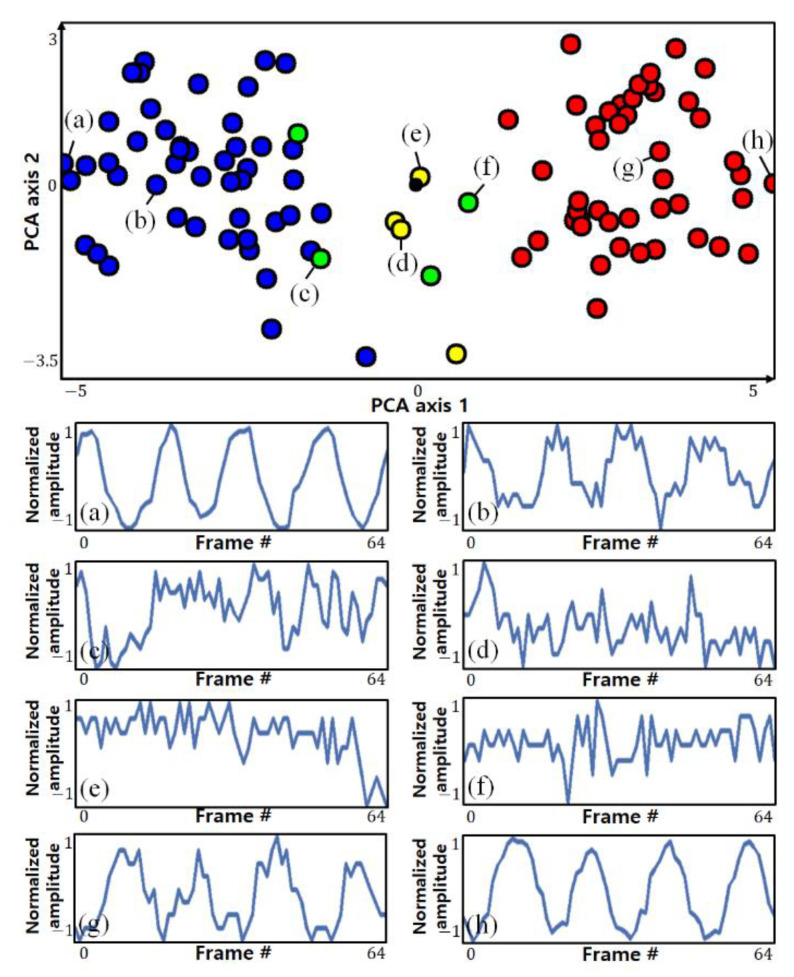
Visualization of the actual ROI pixels and clustering results of PCA (**top**) and the variance in some selected pixels (**bottom**): (**a**–**h**) arbitrary points on a two-dimensional plane and the trajectory of the points (pixels).

Among the pixels including respiration information, in-phase pixels and out-of-phase pixels have characteristics that are symmetrical with respect to the origin. Therefore, the clearer the breathing component is, the more clearly it can be distinguished spatially. Conversely, if the respiratory component is not clear or is close to noise, this symmetry is weak. Using this spatial feature, pixels having similar patterns can be clustered. Figure 4 shows the results of visualizing the pixels and clustering results detected by the actual ROI in two dimensions using principal component analysis (PCA). The circles of the same color indicate pixels classified into the same cluster. The top side of the figure shows each point on a two-dimensional plane, and the bottom side of the figure shows the variance in some selected pixels.

It can be seen that pixels with opposite phases are located at a position symmetrical to the origin, and it can be seen that pixels farther from the origin (with stronger symmetry) show a clearer breathing component. However, it is not possible to know which clusters are clusters with clear respiratory components only from the clustering results, which is an unsupervised learning method, nor can clusters of different phases be distinguished. To make this distinction, it is possible to use the characteristics of the strong symmetry of pixels with distinct respiratory components.

**Figure 5:**
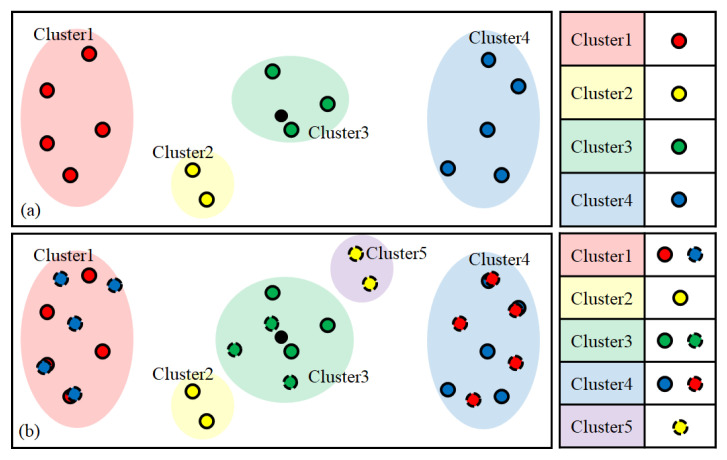
Clustering results of (**a**) original data and (**b**) data including both the original data and symmetric data (dotted circles indicate symmetric data).

For two clusters $C_{1}$ and $C_{2}$ in a symmetrical relationship, if the data in $C_{1}$ are flipped, they are highly likely to be distributed around the location of $C_{2}$, and vice versa. When performing clustering, if not only the original data but also the symmetric data are used together, the two clusters with the strongest mutual inclusion relationship can be regarded as the clusters with the strongest symmetry. Among the clusters of pixels included in the ROI, the two clusters with the strongest symmetry can be assumed to be clusters with clear respiration components and different phases. Figure 5 shows this symmetrical relationship. Figure 5a shows the clustering result of the original data, and Figure 5b shows the clustering result including the symmetrically shifted data. The colored area indicates the cluster including each point, and the table on the right shows the types of data included in each cluster. In Figure 5b, it can be seen that the two clusters 1 and 4, which have strong symmetry, have a mutual inclusion relationship with respect to the symmetrically shifted data.

The method to determine the two clusters with the strongest symmetry is as follows. First, a function *f* for determining whether the *i*-th pixel ($p_{i}$) and its symmetric value ($p_{i}{}^{\prime }$) are included in both groups may be defined as

(3) $$f(n,m,i)=\{\begin{array}{cc} 1 & if\;(p_{i}\in C_{n}\;\;\;and\;\;\;p_{i}{}^{\prime }\in C_{m})\;or\;(p_{i}{}^{\prime }\in C_{n}\;\;\;and\;\;\;p_{i}\in C_{m})\\ 0 & otherwise \end{array}$$

Here, *C* indicates a cluster, where *n* and *m* are the indexes of the two clusters. The two clusters with the strongest symmetry as described by Equation (3) are determined using the following equation:

(4) $$n_{\max},m_{\max}=\underset{n,m}{\arg\max}\sum_{i=1}^{N}f(n,m,i).$$

Here, *N* denotes the number of pixels detected by the ROI.

The respiration component in the video can be estimated by merging the two clusters obtained in this way. In the proposed method, the two clusters are fused simply by inverting the phase of one of the two clusters and obtaining the average of the total. The clustering method uses cosine distance-based hierarchy clustering, which is robust to scale and makes it easy to evaluate the distribution of the data with respect to the origin [22].

## 3. Results

### 3.1. Experimental Setup

Videos used for the training and testing were acquired in RGB format and had a 640 × 480 resolution, 8-bit depth, and 20 fps using a Logitech C920 webcam. The original uncompressed video was saved. The reference signal for respiration was captured simultaneously at 0.01 N resolution and 20 samples per second using a Vernier Go Direct Respiration Belt (GDX-RB). The captured images were reduced by a factor of 8, resampled at 5 fps, and divided into overlapping windows for use as model input. Experimental data were obtained for a total of 15 people (1 training and 14 testing). The research followed the tenets of the Declaration of Helsinki, and informed consent was obtained from the subjects after an explanation of the nature and possible consequences of the study.

**Figure 6:**
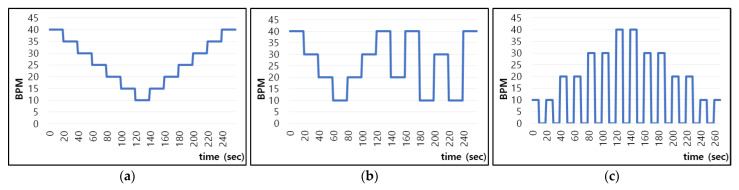
Bpm change in guidelines provided to subjects to acquire training data: (**a**) sequential change in breathing rate; (**b**) rapid change in breathing rate; (**c**) patterns involving apnea.

The proposed model is designed not to take into account morphological characteristics with individual differences and learns whether the amount of pixel change includes respiration characteristics. Therefore, if only one subject who breathes in various patterns is learned, it can be applied to other people without additional learning. For this reason, we acquired data from one subject for learning and included various breathing patterns, as shown in Figure 6.

**Figure 7:**
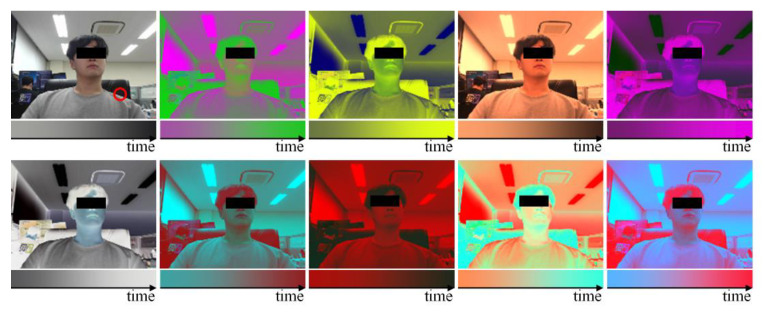
Examples of augmented data used for training.

The subject breathed according to the respiration signal guidelines of the pattern in which breathing changes by 5 sequentially within 10–40 bpm every 20 s, as shown in Figure 6a, the pattern in which breathing changes rapidly up to 30 bpm every 20 s, as shown in Figure 6b, and the pattern in which breathing changes by 10 sequentially within 10–40 bpm every 40 s and includes apnea intervals (10 s) every 10 s. In addition, the training data were augmented to include color information of clothing and background, which are individual characteristics that can influence breathing patterns, by randomly performing scaling, shifting, and inversion for each RGB channel as shown in Figure 7. Figure 7 shows examples of the data actually used for training along with the change over time of a point (red circle) in the video.

**Figure 8:**
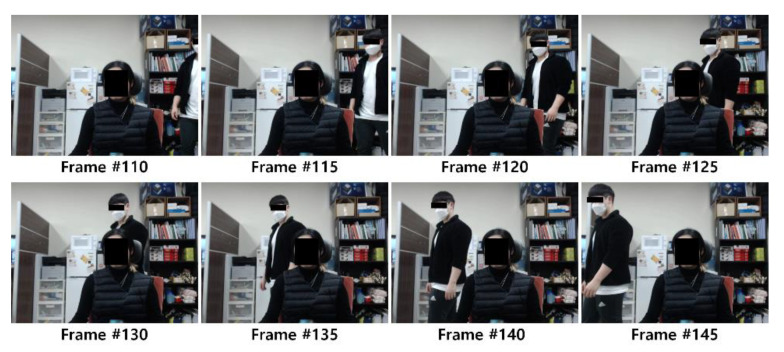
Data samples from Experiment 2 with background noise.

Test data were acquired for a total of 14 people (7 women and 7 men) who wore clothes with various characteristics such as light or dark color. The experiment was designed to capture the subject’s front upper body using a camera installed at a distance of 80 cm from the subject. For the test data, three conditions were captured: a 30 s video without noise in the background while the subject breathed naturally, a 30 s video with a moving object (other subject) in the background while the subject breathed naturally (as shown in Figure 8), and A 70 s video of the subject’s breathing according to the breathing guidelines (in the order of 10, 20, 30, 40, 30, 20, 10 bpm) where the speed changes in sequence every 10 s. These tests are referred to as Experiments 1, 2, and 3, respectively. A total of 10,884 data samples were generated from the videos: 2055, 2049, and 6780 in Experiments 1, 2, and 3, respectively.

The correlation threshold for labeling (*T*) was experimentally defined as 0.7. Training and testing were performed on a notebook equipped with an Intel i7-8750 CPU, 16 GB RAM, GTX 1070, and a 64-bit Windows 10 environment, and these processes were implemented using Python and Keras.

### 3.2. Experimental Results

To evaluate the performance of the proposed method, it was used to acquire the breathing signals from the test videos, which were then compared with the reference signals. The Pearson correlation coefficient (*r*), expressed as correlation, between the two signals (Equation (1)) was used for comparison to check whether the estimated signal clearly contains the actual respiration information. The mean absolute error (MAE) was also used to evaluate the error between the bpm and peak-to-peak interval (PPI) calculated in the estimated and reference signals. A simple method using the PPI was used to calculate bpm. The mean of differences (MOD) and limits of agreement (LOA), defined as ± standard deviation × 1.96, of Bland–Altman plot analysis were also obtained, and the coefficient of determination (expressed as *R*^2^) was used to analyze the degree of agreement between the two measurements.

**Figure 9:**
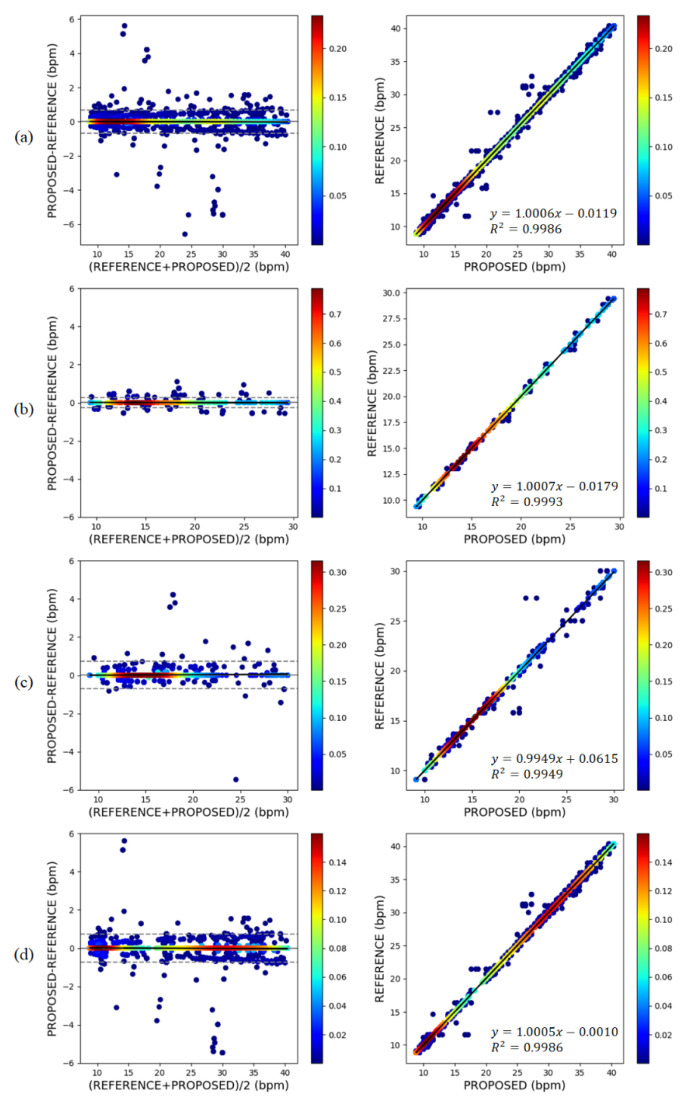
Bland–Altman plot and regression analysis results for bpm: (**a**) overall data; (**b**) Experiment 1; (**c**) Experiment 2; (**d**) Experiment 3.

Table 1 summarizes the measurement performance of the proposed method for each experiment, and Figure 9 shows the Bland–Altman plots and regression analysis results. For the entire data, the Pearson correlation between the estimated respiration signal and the reference was 0.93 ± 0.10, showing a high degree of similarity. The MAE of the bpm calculated from the estimated signal was 0.09 ± 0.33, the MOD was −0.0011, and the LOA was ±0.6678, indicating that the proposed respiration estimation method achieved very satisfactory performance. Figure 9 visualizes the density of the points using kernel density estimation. In all experiments, it can be seen that most of the points are very dense at zero. Because a distribution bias according to changes in the bpm change is not observed, it can be inferred that the performance is consistent with respect to the respiratory rate. In addition, in the regression analysis chart, a very high correlation can be confirmed when the slope of the regression line is very close to 1 and *R*^2^ is 0.99 or more.

**Figure 10:**
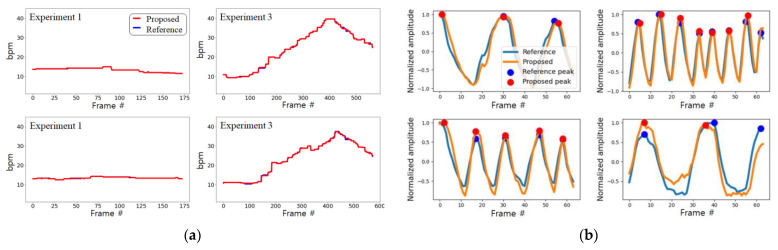
Examples of bpm estimation results and peak detection results in Experiments 1 and 3: (**a**) change in bpm measurement; (**b**) examples of peaks detected in the actual signal.

The performance for Experiment 1, in which there is no background noise and consistent breathing is maintained, yielded the highest performance in all areas. In contrast, Experiment 2 yielded the lowest values for MAE, MOD, and LOA (0.09 ± 0.36, 0.0248, and ±0.7195, respectively). However, these values are still very good given that the defined normal breathing range is 10–40 bpm. Figure 10 shows actual samples comparing the reference and the signal measured by the proposed non-contact method.

In the results of Experiment 3, a slight decrease in performance can be observed. In these results, considering that the correlation between the reference and quality of the estimated signal is not substantially different from that in Experiment 1, it is difficult to say that there is a problem in the estimation of the signal. The reason for this difference in performance can be confirmed from the peak detection result in Figure 10b. According to the waveforms, the peak of the signal is detected differently at the beginning and end. This difference in peak detection seems to have influenced the calculation of the bpm in Experiment 3, where the respiratory rate frequently changes. The fact that the MAE of PPI did not increase may be the basis for this. In Figure 10a, it can be seen that the difference in bpm mainly occurs in the section where the respiration rate changes.

**Figure 11:**
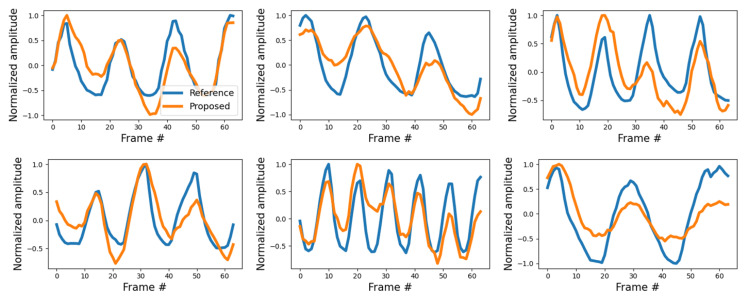
Comparison of signals in Experiment 2.

**Figure 12:**
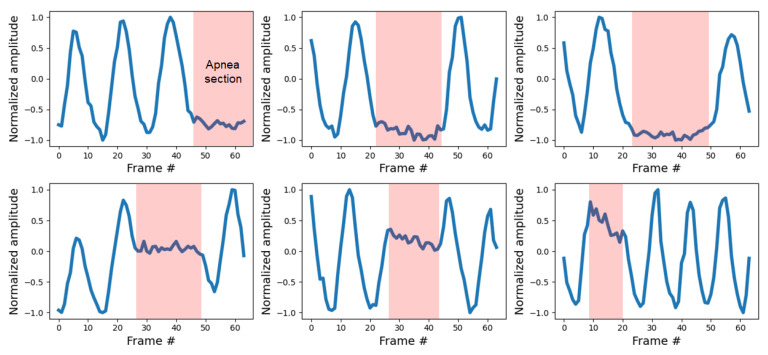
Measurement results of the proposed method for the case where the apnea section is included during breathing.

Figure 11 shows examples of the degraded quality of the estimated signals of Experiment 2. These cases confirm that the signal estimation performance slightly deteriorates in the presence of background noise. However, in most cases, even though the similarity to the reference is lower, this does not substantially affect the estimation of the respiration information. In fact, the performance of Experiment 2 presented in Table 1 shows that the proposed method estimates breathing information without problems despite background noise. A video recording showing the respiratory measurement results of the proposed method for Experiment 2 compared to the reference is given in Supplementary Materials. The proposed method contains information about the apnea interval in the learning, enabling ROI detection even in the apnea interval. Therefore, as shown in Figure 12, the apnea section as well as continuous breathing can be identified.

In order to compare the performance with the proposed method, four studies were confirmed as a result of investigating fully automatic methods similar to our method [14,17,18,19]. However, as the three studies did not present a countermeasure against background noise, it was judged that the comparison with our study was not fair. Therefore, our method was compared with the study of Janssen [19]. As a result of the experiment of this study, *R*^2^ was 0.9905, and considering that the lowest performance of our method was 0.9949, it was confirmed that the numerical performance was better than the previous study. However, since both studies are high above 0.99, it can be judged as a difference that does not have much meaning from the viewpoint of analyzing the interval between peaks of the respiration signal.

In addition, the average processing time and fps for the test data are summarized in Table 2. The total processing time of the proposed method was about 44.5 milliseconds on average, achieving a speed of 22.5 fps.

## 4. Conclusions

We proposed a fully automated respiration measurement method based on commonly used RGB cameras without the need for a separate, expensive device. The proposed method is composed of a method that classifies pixels containing respiration information based on deep learning, and a method that estimates which pixels contain clear respiration information using symmetry. The proposed method achieved a real-time performance of 20 fps in the test environment, and it was evaluated through videos and reference signals acquired in a real environment. The results confirm that the MAE between the estimated signal and the signal of the contact respiration measuring device was very high (approximately 0.09). In addition, the correlation coefficient between the contactless signal and reference signal was 0.93 on average, which confirms that the similarity between the two signals is very high. Several cases demonstrated that the quality of the estimated signal is degraded when the motion noise is severe, but the remaining signal is still suitable for measuring respiration information. However, it is a clear limitation that still exists in our method that the ROI cannot be detected when the subject to be measured moves, and this should be improved in order to be used universally. We plan to improve the breathing measurement for subjects that move instead of remaining stationary in future studies. In addition, in future studies, we will improve the respiration measurement performance and improve stability through model optimization and algorithm improvement while maintaining a low computational cost, and we will consider how to measure the individual breathing of two or more subjects. In peak detection for bpm calculation, verification of which method is suitable for respiratory measurement is also planned. In addition, the performance of the proposed method will be verified in detail for various breathing patterns including apnea patterns.

## Figures and Tables

**Table 1:** Performance results for the overall data and each of the three experiments.

Data	Correlation (bpm)	*R*^2^ (bpm)	MOD (bpm)	LOA (bpm)	MAE (bpm)	MAE (PPI)
All	0.926	0.9986	−0.001	±0.67	0.089	91 ms
Experiment 1	0.936	0.9993	0.006	±0.27	0.045	98 ms
Experiment 2	0.888	0.9949	0.025	±0.72	0.093	120 ms
Experiment 3	0.934	0.9986	−0.011	±0.73	0.101	84 ms

**Table 2:** Performance results for the overall data and each of the three experiments.

Processing Step	Processing Time (ms)	Processing Speed (fps)
Detecting ROI	41.1	24.4
Estimating respiration	3.4	295.4
Total	44.5	22.5

## Data Availability

Data cannot be shared because private information such as faces is included in the data, and an experimental consent has been obtained that will be used only in this study.

## Supplementary Materials

The following are available online at https://www.mdpi.com/article/10.3390/s21103456/s1, Video S1: A video record showing the breath measurement results of the proposed method compared to a reference in a situation with noise in the background (Experiment 2).

## References

[1] Ponikowski P.P., Chua T.P., Francis D.P., Capucci A., Coats A.J., Piepoli M.F. (2001). Muscle Ergoreceptor Overactivity Reflects Deterioration in Clinical Status and Cardiorespiratory Reflex Control in Chronic Heart Failure. Circulation.

[2] Maharaj R., Ivan R., Julia W. (2015). Rapid response systems: A systematic review and meta-analysis. Crit. Care.

[3] Rambaud-Althaus C., Althaus F., Genton B., D’Acremont V. (2015). Clinical features for diagnosis of pneumonia in children younger than 5 years: A systematic review and meta-analysis. Lancet Infect. Dis..

[4] Hogan J. (2006). Why don’t nurses monitor the respiratory rates of patients?. Br. J. Nurs..

[5] Liu H., Allen J., Zheng D., Chen F. (2019). Recent development of respiratory rate measurement technologies. Physiol. Meas..

[6] Hu M.-H., Zhai G.-T., Li D., Fan Y.-Z., Chen X.-H., Yang X.-K. (2017). Synergetic use of thermal and visible imaging techniques for contactless and unobtrusive breathing measurement. J. Biomed. Opt..

[7] Cho Y., Julier S.J., Marquardt N., Bianchi-Berthouze N. (2017). Robust tracking of respiratory rate in high-dynamic range scenes using mobile thermal imaging. Biomed. Opt. Express.

[8] Hu M., Zhai G., Li D., Li H., Liu M., Tang W., Chen Y. (2018). Influence of image resolution on the performance of remote breathing rate measurement using thermal imaging technique. Infrared Phys. Technol..

[9] Van Gastel M.M., Stuijk S.S., De Haan G.G. (2016). Robust respiration detection from remote photoplethysmography. Biomed. Opt. Express.

[10] Zhao F., Li M., Qian Y., Tsien J.Z. (2013). Remote Measurements of Heart and Respiration Rates for Telemedicine. PLoS ONE.

[11] Nam Y., Lee J., Chon K.H. (2013). Respiratory Rate Estimation from the Built-in Cameras of Smartphones and Tablets. Ann. Biomed. Eng..

[12] Reyes B.A., Reljin N., Kong Y., Nam Y., Chon K.H. (2016). Tidal Volume and Instantaneous Respiration Rate Estimation using a Volumetric Surrogate Signal Acquired via a Smartphone Camera. IEEE J. Biomed. Health Inform..

[13] Massaroni C., Presti D.L., Formica D., Silvestri S., Schena E. (2019). Non-Contact Monitoring of Breathing Pattern and Respiratory Rate via RGB Signal Measurement. Sensors.

[14] Bartula M., Tigges T., Muehlsteff J. Camera-based system for contactless monitoring of respiration. Proceedings of the 2013 35th Annual International Conference of the IEEE Engineering in Medicine and Biology Society (EMBC).

[15] Wiede C., Richter J., Manuel M., Hirtz G. Remote Respiration Rate Determination in Video Data-Vital Parameter Extraction based on Optical Flow and Principal Component Analysis. Proceedings of the 13th International Joint Conference on Computer Vision, Imaging and Computer Graphics Theory and Applications.

[16] Ganfure G.O. (2019). Using video stream for continuous monitoring of breathing rate for general setting. Signal Image Video Process..

[17] Tan K.S., Saatchi R., Elphick H., Burke D. Real-time vision based respiration monitoring system. Proceedings of the 2010 7th International Symposium on Communication Systems, Networks & Digital Signal Processing (CSNDSP).

[18] Li M.H., Yadollahi A., Taati B. A non-contact vision-based system for respiratory rate estimation. Proceedings of the 2014 36th Annual International Conference of the IEEE Engineering in Medicine and Biology Society.

[19] Janssen R., Wang W., Moço A., De Haan G. (2015). Video-based respiration monitoring with automatic region of interest detection. Physiol. Meas..

[20] Brox T., Bruhn A., Papenberg N., Weickert J. (2004). High Accuracy Optical Flow Estimation Based on a Theory for Warping. Proceedings of the Transactions on Petri Nets and Other Models of Concurrency XV.

[21] He K., Zhang X., Ren S., Sun J. Deep residual learning for image recognition. Proceedings of Proceedings of the IEEE Conference on Computer Vision and Pattern Recognition.

[22] Johnson S.C. (1967). Hierarchical clustering schemes. Psychometrika.

